# Development and Characterization of an Endotoxemia Model in Zebra Fish

**DOI:** 10.3389/fimmu.2018.00607

**Published:** 2018-03-29

**Authors:** Alan Y. Hsu, Theodore Gurol, Tiago J. P. Sobreira, Sheng Zhang, Natalie Moore, Chufan Cai, Zhong-Yin Zhang, Qing Deng

**Affiliations:** ^1^Department of Biological Sciences, Purdue University, West Lafayette, IN, United States; ^2^Bindley Bioscience Center, Purdue University, West Lafayette, IN, United States; ^3^Purdue Institute for Drug Discovery, Purdue University, West Lafayette, IN, United States; ^4^Department of Medicinal Chemistry and Molecular Pharmacology, Purdue University, West Lafayette, IN, United States; ^5^Purdue Institute for Inflammation, Immunology, and Infectious Disease, Purdue University, West Lafayette, IN, United States; ^6^Purdue University Center for Cancer Research, Purdue University, West Lafayette, IN, United States

**Keywords:** endotoxin, lipopolysaccharide, endotoxemia, zebra fish, innate immunity, inflammation

## Abstract

Endotoxemia is a condition in which endotoxins enter the blood stream and cause systemic and sometimes lethal inflammation. Zebra fish provides a genetically tractable model organism for studying innate immunity, with additional advantages in live imaging and drug discovery. However, a *bona fide* endotoxemia model has not been established in zebra fish. Here, we have developed an acute endotoxemia model in zebra fish by injecting a single dose of LPS directly into the circulation. Hallmarks of human acute endotoxemia, including systemic inflammation, extensive tissue damage, circulation blockade, immune cell mobilization, and emergency hematopoiesis, were recapitulated in this model. Knocking out the adaptor protein Myd88 inhibited systemic inflammation and improved zebra fish survival. In addition, similar alternations of pathways with human acute endotoxemia were detected using global proteomic profiling and MetaCore™ pathway enrichment analysis. Furthermore, treating zebra fish with a protein tyrosine phosphatase nonreceptor type 11 (Shp2) inhibitor decreased systemic inflammation, immune mobilization, tissue damage, and improved survival in the endotoxemia model. Together, we have established and characterized the phenotypic and gene expression changes of a zebra fish endotoxemia model, which is amenable to genetic and pharmacological discoveries that can ultimately lead to a better mechanistic understanding of the dynamics and interplay of the innate immune system.

## Introduction

The clinical manifestations associated with Gram-negative bacterial infection, such as vascular damage and leakage, disseminated intravascular coagulation, tissue hypoxia, systemic inflammation, cytokine storm, and in extreme cases elevation into sepsis (1, 2), are often caused by the presence of bacteria cell wall contents. Principally among these components are endotoxins or lipopolysaccharides (LPS) (3), which enter the blood circulation (4). A reductionist approach is taken to obtain a clean understanding of the host response elicited upon recognizing endotoxins in cell cultures and mice models, leaving out the complex interactions associated with tissue damage caused by live bacteria (5). Cultured cells offer a potent platform to dissect the signaling pathways activated by an LPS stimulation, but not the interactions of different cell types *in vivo*. Mice models have offered many significant insights at the whole organism level in response to endotoxemia yet suffer from the throughput, as well as divergence in the altered pathways (6). As such, there is a need to develop other animal models that can complement the cell culture and murine models.

The zebra fish is a fully sequenced model organism (7) with a highly conserved innate immune system, including cell types and signaling molecules (8, 9). Zebra fish larvae develop innate immune cells within 2 days post fertilization (dpf) while the adaptive immune system is not present until approximately 3 weeks post fertilization (10, 11), making it a favorable model to study innate immune-dominant responses. The transparent nature of zebra fish larvae allows imaging of dynamic interactions between immune cells and somatic tissues at the whole organism level (8, 12). Additional advantages of using zebra fish include high fecundity and ease of gene editing, making it an ideal model for high-throughput genetic and compound screening (13).

Despite a lack of a complete understanding of the LPS receptor in zebra fish (14), the downstream immune adaptors, pathways, and general immune response to LPS (9, 15) are highly conserved. It is expected that a zebra fish endotoxemia model will advance our understanding of the initiation and resolution of the systemic inflammation during acute endotoxemia in humans.

Lipopolysaccharide has previously been used as an immune-stimulant to cause inflammation in zebra fish larvae and to screen for anti-inflammatory compounds, through injection into the yolk (16) or bathing of the whole larvae. A single injection of 1 ng LPS into the yolk of 3 dpf zebra fish larvae caused upregulation of pro-inflammatory cytokines and caused 100% lethality at 24 hpi. The limitation of this approach is a lack of yolk equivalent tissue in humans and poor physiological conservation (16). The LPS bath model has been used in several studies (12, 17, 18), where vascular damage/leakage, tail fin edema, and immune activation were observed. However, the constant presence of LPS in the bath causes an overwhelming systemic inflammation that initiates at the epithelial surface and does not allow the detoxification of LPS and subsequent resolution of the inflammation (12, 17, 18). The pivotal role of immunostimulant introduction has been previous reported where bacterial injection and emersion caused dysregulation of overlapping but distinct responsive genes (19). Furthermore, an administration route that faithfully mirrors human immune interactions is necessary to facilitate a true endotoxemia model (13). Hence, a *bona fide* zebra fish endotoxemia model still needs to be developed.

We have designed a 3D-printable injection plate to facilitate a higher throughput for zebra fish larvae intravenous (IV) injection. Delivering LPS into the blood stream leads to acute systemic inflammation that starts to resolve at 24 hpi. Conserved phenotypic and gene expression changes are also observed in our model. Together, we have developed and characterized a zebra fish endotoxemia model that represents the fundamental biological processes of endotoxemia in humans. This model can be used in combination with murine models to fully dissect the molecular mechanisms regulating the magnitude of inflammation and to discover compounds that would mitigate detrimental consequences in the host.

## Materials and Methods

### Fish Husbandry

This study was carried out in accordance with the recommendations of “Use of Zebrafish in the NIH Intramural Research Program.” The Animal Care and Use Protocol was approved by The Purdue Animal Care and Use Committee (PACUC) (Protocol number: 1401001018). The transgenic lines *Tg(NF-kB:GFP)* (20), *Tg(mpx:mcherry)* (21), and *Tg(mpeg:EGFP-H2B)* (22) were previously described. SecAV-YFP was PCR amplified using the forward: 5′- TATAGGGCGAATTGGGTACCGCCACCATGCATAAGGTTT-3′ and reverse: 5′- ACCGCGGTGGCGGCCGCTTACTTGTACAGCTCGTCC-3′ from pBH-UAS-secA5-FP (Addgene plasmid #32359) and inserted into the KpnI/NotI cloning site of pMe Gateway plasmid (Invitrogen). A three-way LR reaction with p5e-βactin, pMe-SecAV-YFP, and p3e-SV40polyA was performed in pDestR4R3 backbone to obtain pTol2-βactin:SecAV-YFP. The plasmids will be deposited to Addgene before acceptance of the paper. More than three founders (F0) for *Tg(*β*actin:SecAV-YFP)^*pu17*^* were obtained as described (23). All experiments were performed with embryos at 3 dpf unless noted otherwise.

### Injection Plate Design

A 3D printer (Model Ultimaker 2+) was used to print the mold from an autoCAD schematic drawing with solid filling at 150-µM resolution. The mold is a negative of a 10-cm Petri dish with the upper portion resting on the walls of the dish; 3% Agar/E3 media were heated and poured directly into the Petri dish, and the mold was directly layered on the top. The mold was then removed from the Petri dish and the injection plate was ready for use.

### Microinjection

All injections were performed by microinjection. Embryos were collected before the first cell division and injected directly into the cell. Injection needles (Warner instruments 6100TF-3) were pulled with P-1000 micropipette puller (Sutter instruments) using program #37 with ramp = 540. Embryos were placed under a Leica M125 dissection microscope. Microinjections were performed with a picospritzer II (Parker Hannifin) with an output pressure of 30 psi and a pressure duration between 20 and 30 ms. Injection volume was calibrated using volume = 4/3 π*r*^3^, where a radius (*r*) of 5 µm gives a volume of 1 nl. To perform blood injection, staged larvae were placed in the injection mold under an Olympus SZ61 dissection microscope. Injection needles (Sutter instruments BF100-5810) were pulled with P-1000 micropipette puller (Sutter instruments) using program #34 with ramp = 570. Injection was performed with a picospritzer III (Parker Hannifin) with an output pressure at 40 psi and a pressure duration between 30 and 50 ms. Injection volume was calibrated and set to 1 nl as described above.

### Survival Assay

Larvae were injected with 1 nl of 25 ng/nl LPS (Sigma L9143) into the tail vein and incubated individually in 96-well plates. Survival was tracked for 5 days or when one group reached 100% mortality. Representative experiments of at least three independent repeats (*n* = 20 larvae in each experiment) were shown.

### Proteomic Analysis

For global proteomic analysis, 40 larvae at 3 dpf were injected with PBS or LPS IV, with 40 uninjected larvae as control. At 8- and 24-h post injection (hpi), 20 larvae each were deyolked and frozen at −80^°^C and sent for proteomic analysis. Samples from three individual repeats were collected, and the global proteomics profiling and downstream statistical analysis were performed by the Purdue Proteomics Facility. The results from the mass spectrometer were processed using the MaxQuant computational proteomics platform version 1.5.8.3 (24). The peak list generated was searched against the *Danio rerio* sequences from UNIPROT retrieved on April 27, 2017, and a common contaminants database. The following settings were used for MaxQuant: default Orbitrap parameters, minimum peptide length of seven amino acid, data were analyzed with “Label-free quantification” (LFQ) checked and the “Match between runs” interval set to 1 min, protein FDR was set to 1%, enzyme trypsin and LysC allowing for two missed cleavage and three modifications per peptide, fixed modifications were carbamidomethyl (C), variable modifications were set to Acetyl (Protein N-term) and Oxidation (M). An in-house script was used to perform the following steps on the MaxQuant results: removal of all the common contaminant proteins, log-transformed [log_2_(*x*)] the LFQ intensity values, input the missing values using the average values of the other two samples when just one sample was missing, and use half of the lowest intensity when all three samples were missing in one group and presented in all three samples in the other group. The heat maps and statistical analyses were performed in the R-environment (www.cran.r-project.org). A *t*-test was performed on the LFQ intensities, and only proteins with a *p-value* of <0.05 and a fold change greater than 1.5 or less than −1.5 were used for the heat map and pathway analyses. The pathway analysis was conducted with MetaCore™ version 6.30 build 68,780, and the selected genes were mapped to the *Homo sapiens* pathways. Proteomic data were deposited to Mass Spectrometry Interactive Virtual Environment (MassIVE) at UCSD. MassIVE ID: MSV000081612 (ftp://MSV000081612@massive.ucsd.edu provisory Username: MSV000081612 Password: hsualan2017; these data will be freely available after publication).

### Terminal Deoxynucleotidyl Transferase-Mediated dUTP-Biotin Nick End Labeling (TUNEL) and Acridine Orange (AO) Staining

Terminal deoxynucleotidyl transferase-mediated dUTP-biotin Nick End Labeling staining was performed as described (23). Briefly, embryos were fixed in 4% paraformaldehyde in phosphate-buffered saline for 2 h and stored in 100% methanol overnight before rehydration and staining with TUNEL label and enzyme Mix (Roche). AO staining was performed by incubating larvae in the dark for 30 min with 5 µg/ml AO (Sigma A6014), followed by washing with fresh E3 for at least five times and visualization with the GFP channel.

### Live Imaging and Image Quantification

Larvae at 3 dpf were placed on a glass-bottomed dish in E3 media containing 0.02% tricaine (Sigma). Representative images were taken with AXIO Zoom V16 microscope (Zeiss) at 70% magnification (zoom 7) at the trunk region in AO and SecAV-YFP imaging, 100% (zoom 10) magnification for TUNEL imaging, and 25% (zoom 2.7) for whole-body imaging. All images were taken with the same exposure to avoid saturation of the CCD detector. Images were processed with Image J by background subtraction with the rolling ball radius as 50 and then quantified for signal intensity in the region of interest. For quantification of fluorescence-labeled neutrophils and macrophages, cells were counted blindly in the indicated caudal hematopoietic tissue (CHT) regions at the designated time points. Each experiment included at least 20 zebra fish larvae and was independently repeated three times. Graphs were generated using PRISM 6 (GraphPad).

### Reverse Transcription-Quantitative PCR (qPCR)

Total RNA was extracted using RNeasy RNA purification kit (Qiagen). Messenger RNAs were reverse-transcribed with Transcriptor First Strand cDNA Synthesis Kit (Roche). qPCR were performed with the FastStart Essential DNA Green Master (Roche). Primers are listed in Table S1 in Supplementary Material. All primers amplified a single product according to the melt-curve analysis. The relative fold change is calculated following instructions provided by real-time PCR Minor with correction of the primer efficiencies (http://ewindup.info/miner/data_submit.htm). At least 20 larvae were used in each biological replicate to generate an average value that was used to calculate the final mean ± SD from three independent experiments.

### Knockout With CRISPR/Cas9

*Myd88* knockout was performed as previously described (25). Briefly, sgRNAs against MyD88 and RFP without off-targeting were selected using CRISPRScan (26). Two individual sgRNAs were synthesized for *myd88* and *rfp*. Solution of 1 nl containing 400 ng/µl sgRNAs and 400 ng/µl Cas9 protein (PNA, CP01) was injected into the one-cell stage of fertilized embryos. No development abnormality was observed at 3 dpf (25). Primers used to synthesize templates of sgRNAs are listed below from 5′ to 3′: MyD88 sgRNA1 forward: TAATACGACTCACTATAGGCGGCAGACTGGAGGACAGGTTTTAGAGCTAGAAATAGCAAG; MyD88 sgRNA2 forward: TAATACGACTCACTATAGGAAAAGGTCTTGACGGACTGTTTTAGAGCTAGAAATAGCAAG; RFP sgRNA1 forward: TAATACGACTCACTATAGGAGGGCTTGCCTTCGCCCTGTTTTAGAGCTAGAAATAGCAAG; RFP sgRNA2 forward: TAATACGACTCACTATAGGTCGGGGATGCCCTGGGTGGTTTTAGAGCTAGAAATAGCAAG. The reverse primer for all sgRNA templates is 5′-AAAAGCACCGACTCGGTGCCACTTTTTCAAGTTGATAACGGACTAGCCTTATTTTAACTTGCTATTTCTAGCTCTAAAAC-3′.

### Anti-Inflammatory Chemical and Drug Treatment

Larvae at 3 dpf were incubated in dexamethasone (sigma) and hydrocortisone (sigma) at a final concentration at 100 µM, in 1% DMSO in E3 as previously reported (27), or with 0.6 µM (3ki) Shp2 inhibitor 11a-1 (28). At the specified concentrations, no development abnormality was observed.

### Statistical Analysis

Statistical analysis was carried out using PRISM 6 (GraphPad) and Mann–Whitney test (comparing two groups), and Kruskal–Wallis test (when comparing to a single group). For qPCR, each gene was normalized to the reference gene *ef1α* and compared with Sidak’s multiple comparison test for gene panels and Mann–Whitney test for single comparisons. For survival assays, Gehan–Breslow–Wilcoxon test was performed with a log-rank test and confirmed with Kaplan–Meier curve to ensure compatibility.

## Results

### Establishment of an Endotoxemia Model

**Figure 1:**
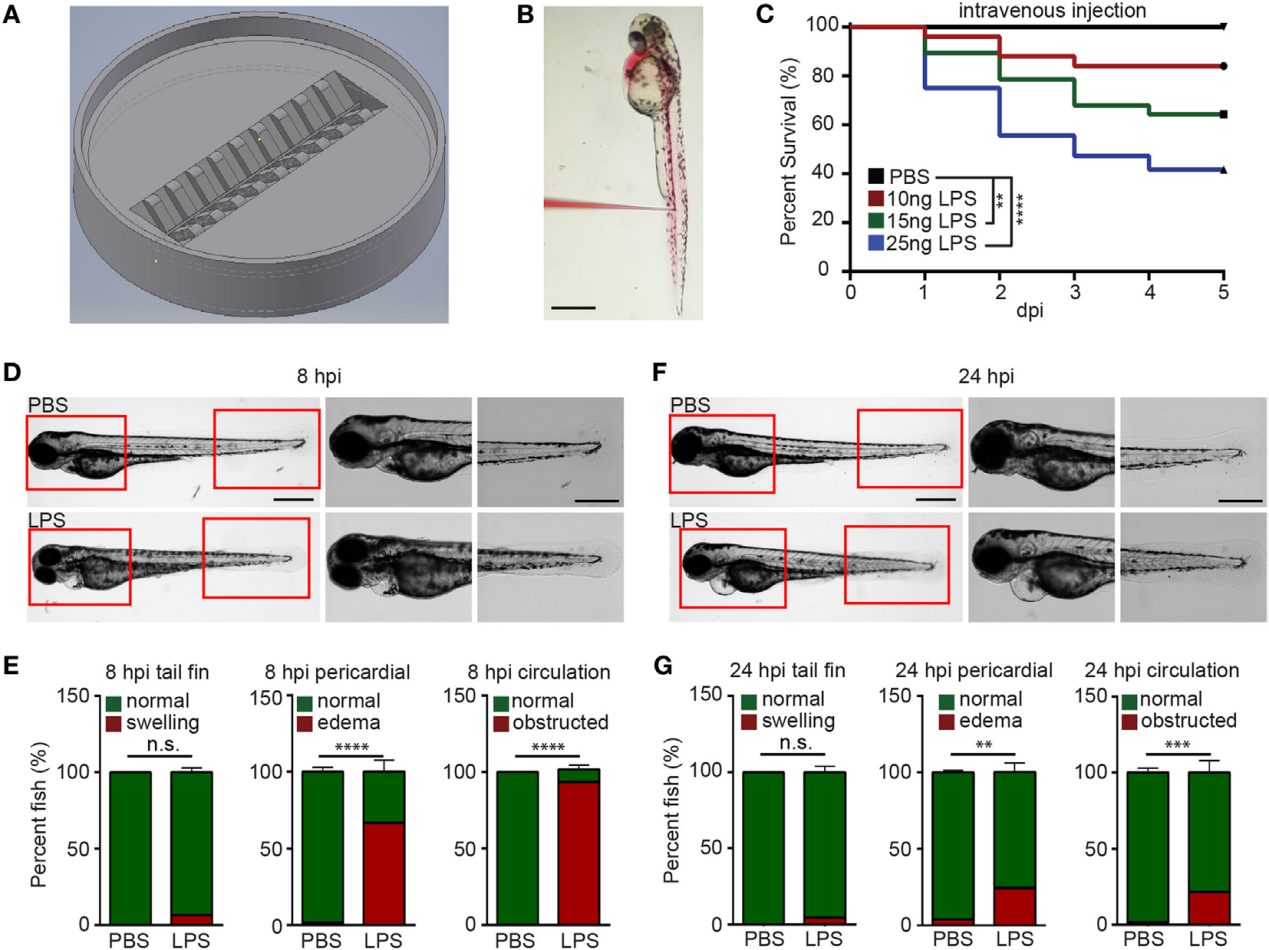
Zebra fish intravenous (IV) injection plate design and IV lipopolysaccharide (LPS) injection. **(A)** Illustration of the zebra fish IV injection plate. **(B)** A representative image of zebra fish larvae at 3 days post fertilization injected with PBS containing 1% Phenol Red. Scale bar: 500 µm. **(C)** Survival curve of zebra fish after PBS or 10, 15, and 25 ng LPS IV injection. One representative experiment of three independent biological repeats (*n* > 20 each group) is shown. **p < 0.01, ****p < 0.0001, Gehan–Breslow–Wilcoxon test. **(D)** Representative images of zebra fish larvae injected with PBS or LPS at 8 hours post injection (hpi). Scale bar: 500 µm. Boxed regions of the pericardium and tail fin are enlarged. Scale bar: 200 µm. Images representative of three independent experiments are shown (*n* = 20). **(E)** Percentage of fish with tail fin edema, pericardial edema, and circulation obstruction in PBS- or LPS-injected larvae at 8 hpi. Results are presented as mean ± SD (*n* = 3 independent experiments with over 20 larvae each/experiment). ***p < 0.001, ****p < 0.0001, Mann–Whitney test. **(F,G)** Representative images and quantification of inflammatory phenotypes in LPS- or PBS-injected larvae as **(D,E)** at 24 hpi.

To efficiently inject LPS through the IV route, we adapted a previously reported method (29) and further optimized it by positioning larvae in an injection plate custom-made with a 3D-printed mold (Figures 1A,B; Figure S1 in Supplementary Material and 3D printing file). This plate can hold 20 zebra fish larvae in individual clefts and allows the injection of 1–5 dpf zebra fish larvae into any position in the trunk. Utilizing this setup, we were able to inject 3 dpf larvae IV at a rate of 150 larvae/h. With any immune process, the dose and duration of challenge greatly dictates the immune response and phenotype (30, 31). LPS from *Pseudomonas aeruginosa* was selected because of its virulence in the bath model (17), and in addition, the *Pseudomonas* species are a prevalent cause of infections and acute inflammation in intensive care units (32). The injection of 10, 15, and 25 ng LPS induced a dose-dependent mortality ranging from 20 to 60% at 5 days post injection (Figure 1C). Control larvae injected with PBS did not show any mortality. As such, a dose of 25 ng, reproducibly causing approximately 50% mortality, was selected for future experiments. At 8 hpi, the majority of LPS-injected larvae exhibited pericardial edema and circulation obstruction (Figures 1D,E; Movie S1 in Supplementary Material). However, only a few larvae displayed tail fin abnormalities, in contrast to the observation made when bathing the fish with LPS (18). At 24 hpi, the percentage of pericardial edema and circulation obstruction in LPS-injected larvae group decreased, suggesting a recovery in some of the larvae that have survived the acute challenge (Figures 1F,G; Movie S2 in Supplementary Material) despite some larvae still having abnormalities. Our model coincides with pulmonary edema and circulatory obstruction which have been reported to be outcomes of acute endotoxemia in humans (33, 34).

### Systemic NF-κB Activation in the Endotoxemia Model

**Figure 2:**
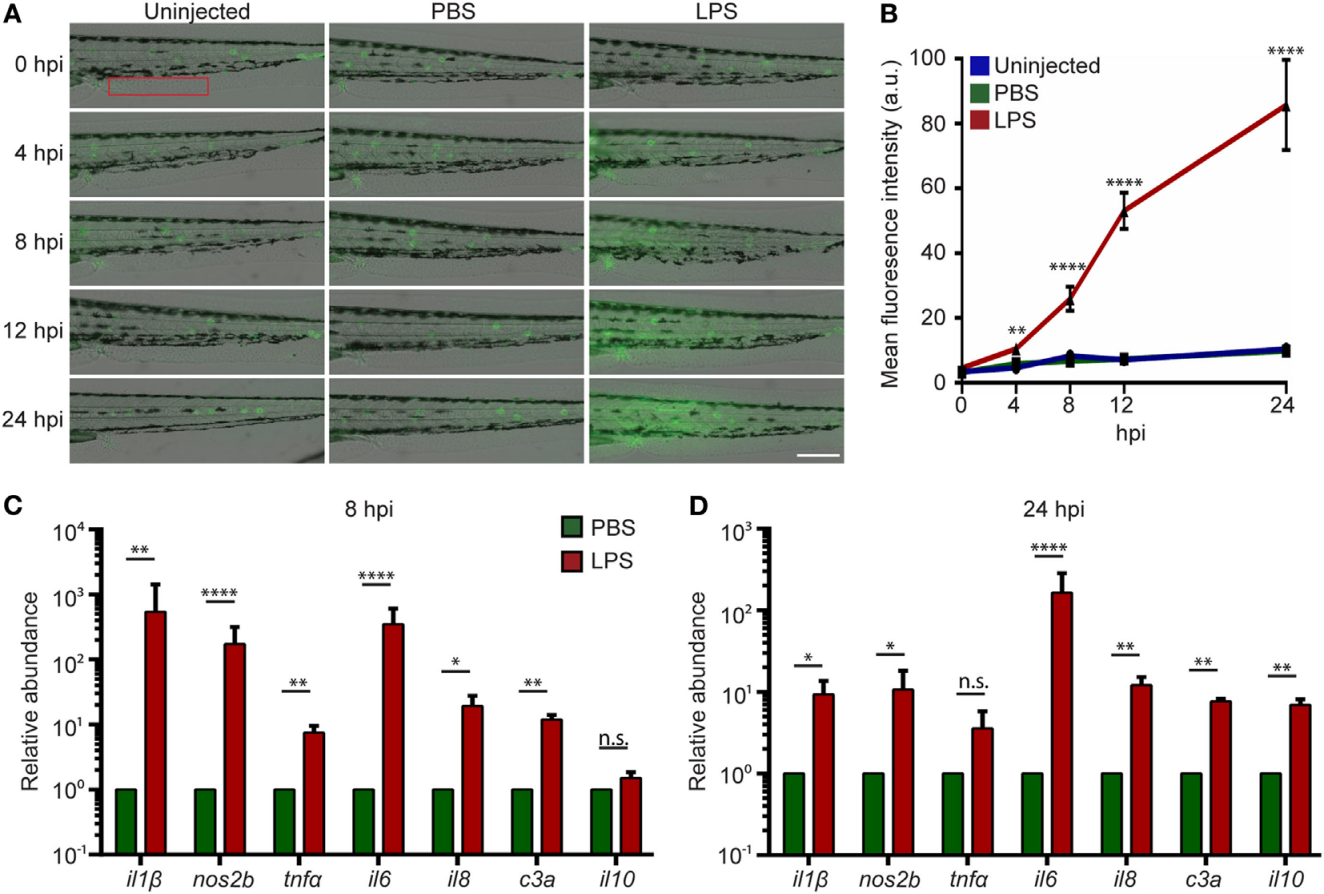
Intravenous (IV) lipopolysaccharide (LPS) activates NF-κB pathway and elicits a systemic immune response. **(A)** *Tg(NF:*κ*B:GFP)* larvae were IV injected with PBS, LPS, or left uninjected. Representative images of GFP signals at indicated time points post injection. Images representative of three independent experiments were shown (*n* = 20). Scale bar: 200 µm. **(B)** Quantification of the GFP intensity in the caudal fin region (red box in Figure 2A). Results are presented as mean ± SD **(D)** (*n* = 3 independent experiments with over 20 larvae each/experiment). **p < 0.01, ****p < 0.0001, Kruskal–Wallis test. **(C,D)** Transcript levels of genes encoding pro-inflammatory and anti-inflammatory cytokines in whole larvae injected with either PBS or LPS at 8 hpi **(C)** and 24 hpi **(D)**. Results were normalized with *ef1α* and are presented as means ± SD (*n* = 3 biological repeats with 20 larvae in each group). *p < 0.05, **p < 0.01, ***p < 0.001, ****p < 0.0001, Sidak’s multiple comparisons test.

Systemic inflammation is a hallmark of endotoxemia (2, 35) where multiple receptors are activated and signals converge to activate the NF-κB family of transcription factors (36, 37). To determine the degree of inflammation in the whole larvae, an NF-κB reporter zebra fish line, *Tg(NF-*κ*B:GFP)* (38), was used, where the transcription of the GFP reporter gene is activated by NF-κB. Compared to uninjected or PBS-injected controls, LPS-injected larvae exhibited an increase in GFP level throughout the body, indicating systemic NF-kB activation. The tail fin region was selected for quantification, where minimal basal auto-fluorescence was present. GFP intensity significantly increased at 8 and 24 hpi in the LPS-injected larvae (Figures 2A,B). This is consistent with the time frame in acute human endotoxemia when systemic NF-kB and inflammation were noted (39). Furthermore, pro-inflammatory cytokine levels and the complement component *c3a* were significantly upregulated at 8 hpi in endotoxemic larvae compared to PBS-injected controls (Figure 2C), consistent with previous studies (40, 41). This upregulation of pro-inflammatory cytokines and complement component persisted at 24 hpi, though the magnitude decreased significantly, while *il-10*, a signature anti-inflammatory cytokine, was significantly upregulated (Figure 2D) (42), suggesting the initiation of the resolution of the systemic inflammation induced by LPS, at a time point similar to those observed in mice and humans (43–46).

### Tissue Damage in the Endotoxemia Model

**Figure 3:**
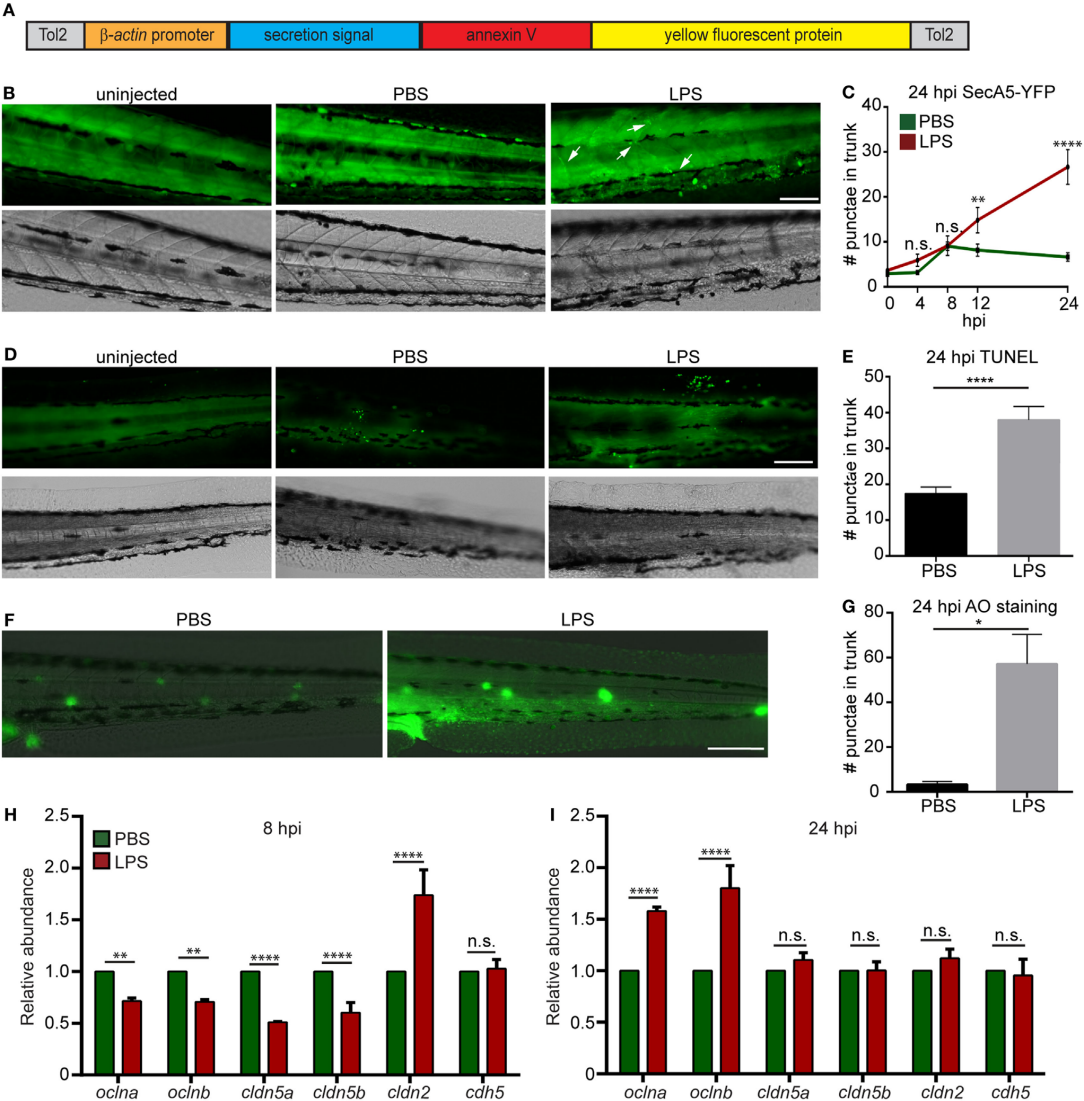
Lipopolysaccharide (LPS) induces tissue damage. **(A)** Diagram of the construct used to generate the apoptosis reporter line where a secreted AnnexinV tagged with a YFP protein is driven by the β*-actin* promoter. **(B,C)** *Tg (*β*-actin:secANV-YFP)* were injected with PBS or LPS. Representative images **(B)** and quantification **(C)** of the YFP-positive foci in trunks at indicated time points post injection. **(D–G)** WT zebra fish larvae were injected with PBS or LPS. Representative images **(D)** and quantification **(E)** of terminal deoxynucleotidyl transferase-mediated dUTP-biotin Nick End Labeling (TUNEL)-positive cells in the trunk at 24 hours post injection (hpi). Representative images **(F)** and quantification **(G)** of the Acridine Orange (AO)-positive cells in the trunk at 24 hpi Scale bar: 100 µm. Results are presented as mean ± SEM (*n* = 3 independent experiments with over 20 larvae each/experiment). *p < 0.05, ****p < 0.0001, Mann–Whitney test. **(B,D,F)** Images representative of three independent experiments were shown (*n* = 20). **(H,I)** Transcript levels of genes encoding vascular and cell junction proteins in whole larvae injected with either PBS or LPS at 8 hpi **(H)** and 24 hpi **(I)**. Results were normalized with *ef1α* and are presented as means ± SD **(D)** (*n* = 3 biological repeats with 20 larvae in each group). **p < 0.01, ***p < 0.001, ****p < 0.0001, Sidak’s multiple comparisons test.

To determine the degree of tissue damage in the live animal, we generated a zebra fish line *Tg(*β*actin:secA5-YFP)^*pu17*^*, where AnnexinV tagged with YFP is constitutively produced and secreted (Figure 3A). Apoptotic cells induced with UV are efficiently labeled as originally characterized by van Ham et al. (20) (Figure S2 in Supplementary Material). LPS-injected larvae possessed more AnnexinV puncta in the trunk as well as in the vasculature at 12 and 24 hpi (Figures 3B,C). Two separate approaches, TUNEL (Figures 3D,E) and AO (Figures 3F,G) (47) staining, were used to confirm the results at 24 hpi when the cell death was most prominent. Again, extensive tissue damage, in the vascular tissue and CHT, the bone marrow equivalent in zebra fish larvae, was observed in the LPS-injected group. To further evaluate vascular and cell junction integrity, we measured the mRNA levels of cell junction genes. Occludin members and Claudin-5 genes were significantly downregulated, whereas claudin-2 was significantly upregulated at 8 hpi (Figure 3H), suggesting loss of vascular cell junction integrity (18, 48). Vascular junction genes returned to physiological levels at 24 hpi (Figure 3I) with a higher expression of Occludin family genes which have been shown to facilitate tissue repair (49). These results faithfully recapitulated tissue damage throughout the body which is another hallmark of endotoxemia (1).

### Emergency Hematopoiesis in the Endotoxemia Model

**Figure 4:**
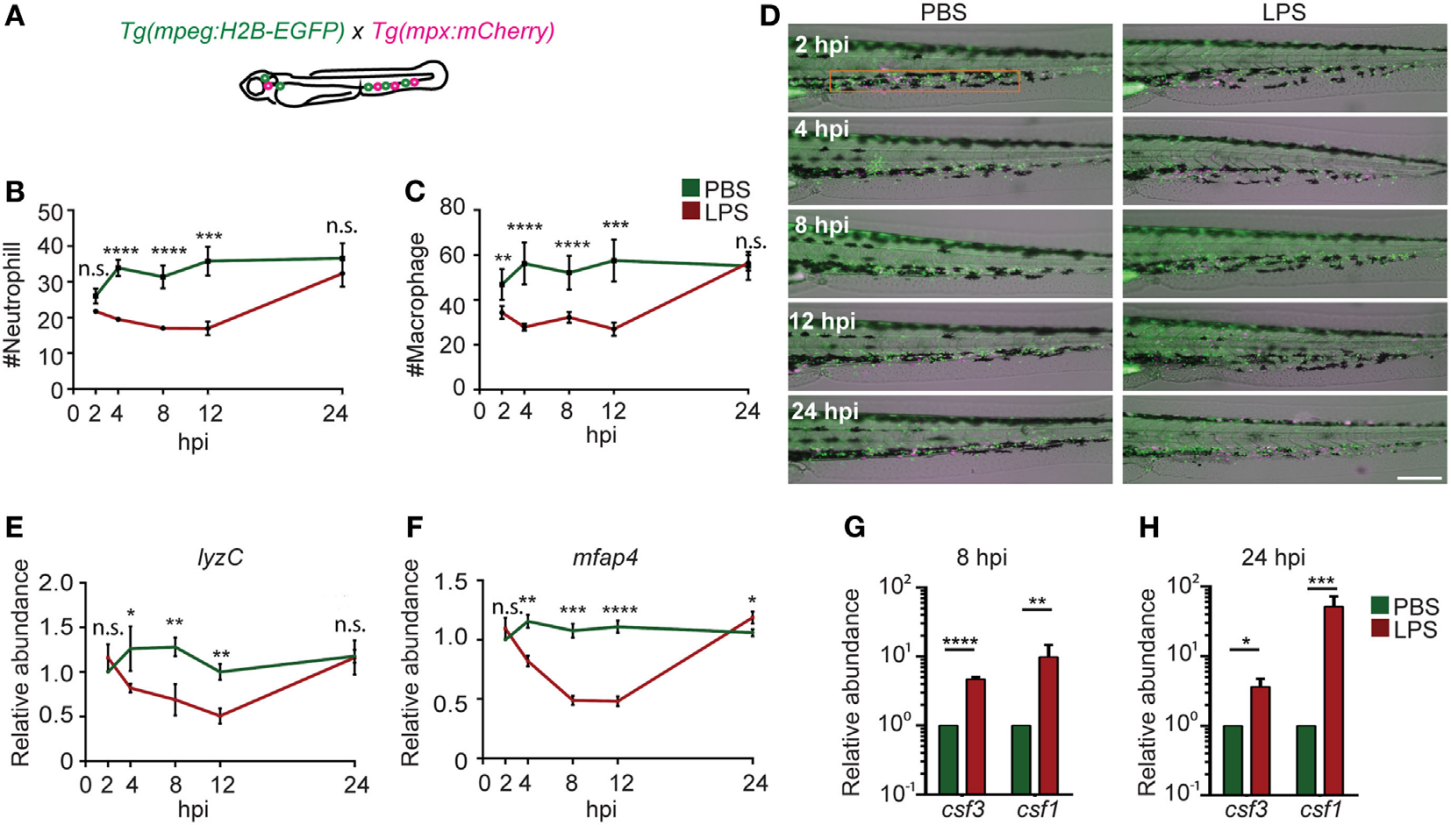
Immune cell mobilization and emergency hematopoiesis induced with lipopolysaccharide (LPS) injection. **(A)**. *Tg(mpeg:H2B-GFP)* fish were crossed with *Tg(mpx:mCherry)* label macrophages (green) and neutrophils (red). The quantification of neutrophil **(B)** and macrophage **(C)** numbers and representative images **(D)** in the caudal hematopoietic tissue (CHT) (orange box) after PBS or LPS injection. Images representative of three independent experiments were shown (*n* = 20). Scale bar: 200 µm. **(E,F)** mRNA level of *lyzC* **(E)** and *mfap4* **(F)** in the whole larvae injected with PBS or LPS. Results were normalized with *ef1α* and are presented as means ± SD **(D)** (*n* = 3 independent experiments with over 20 larvae each/experiment). *p < 0.05, **p < 0.01, ***p < 0.001, ****p < 0.0001, Mann–Whitney test. **(G,H)** mRNA level of *csf3* and *csf1* in whole larvae injected with PBS or LPS at 8 hpi **(G)** and 24 hpi **(H)**. Results were normalized with *ef1α* and are presented as means ± SD (*n* = 3 biological repeats with 20 larvae in each group). *p < 0.05, **p < 0.01, ***p < 0.001, ****p < 0.0001, Mann–Whitney test.

As a result of profound inflammation, high levels of acute phase cytokines such as granulocyte and macrophage colony-stimulating factors, and TNF-α are released into the circulation that mobilize the myeloid cells out of the bone marrow reservoir and induce a rapid differentiation of common myeloid progenitors to replenish the innate immune cells in a process termed “emergency hematopoiesis/granulopoiesis” (50). This phenomenon has been observed during endotoxemia in humanized mice models (51). To determine the kinetics of immune cell mobilization and emergency hematopoiesis, we used a transgenic zebra fish line with macrophages and neutrophils labeled separately, *Tg(mpx:mcherry/mpeg:GFP-H2B)* (Figure 4A) (21, 22). Upon the induction of endotoxemia, neutrophils and macrophages mobilized out of the CHT (orange box region) by 2 hpi and entered the vasculature and the trunk tissue. Immune cells continued to mobilize, and only scarce amounts of phagocytes resided in the CHT at 12 hpi followed by recovery to normal levels at 24 hpi (Figures 4B–D). PBS injection induced a regional inflammatory response which was resolved by 8 hpi and did not induce significant depletion of immune cells in the CHT. For a more quantitative measure in the whole larvae, qRT-PCR for neutrophil (*lyzC*) (52), and macrophage (*mfap4*) (53), specific genes were performed. Similarly, transient declines in both *lyzC* and *mfap4* were noted from 4 to 12 hpi, followed by a full recovery at 24 hpi (Figures 4C,E). Consistently, the hematopoiesis-stimulating growth factors, *csf3 (g-csf)* and *csf1 (m-csf)* (54), were upregulated at both 8 and 24 hpi (Figures 4F,G) as previously reported during emergency hematopoiesis (55). This is the first *in vivo* observation of the dynamics of immune cell mobilization and emergency hematopoiesis in the hematopoietic tissue and at the whole organism level during endotoxemia.

### Myd88 Mediates Inflammation in the Endotoxemia Model

**Figure 5:**
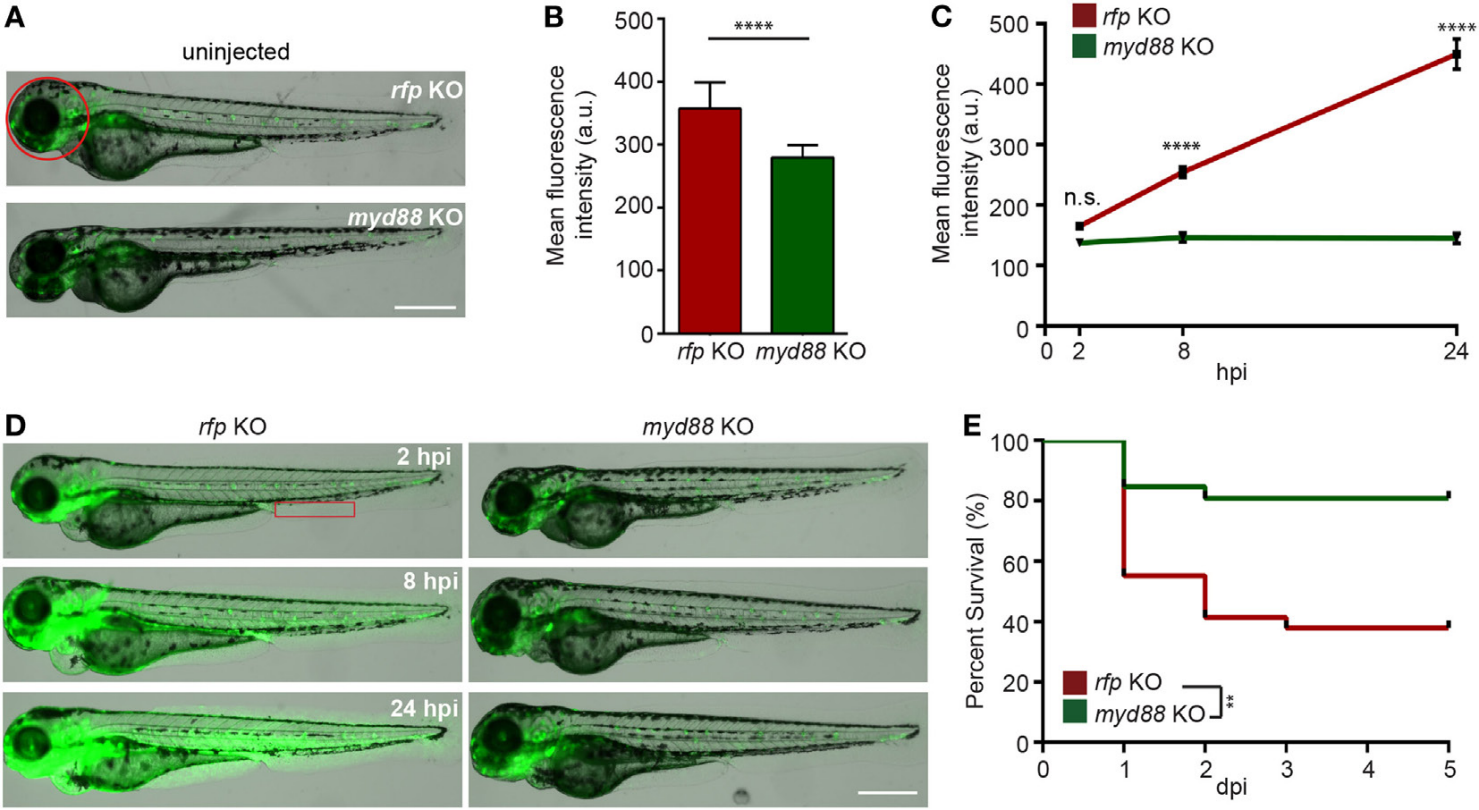
Knockout of MyD88 dampens NF-κB activation and reduced mortality in the zebra fish endotoxemia model. Zebra fish embryos of *Tg(NF:*κ*B:GFP)* were injected with sgRNA against *myd88* or *rfp* at the single-cell stage. **(A,B)** Representative images **(A)** and quantification **(B)** of the GFP intensity in the head (red circle) of uninjected 3dpf larvae. Scale bar: 500 µm. Results are presented as mean ± SD **(D)** (*n* = 3 independent experiments with over 20 larvae each/experiment). ****p < 0.0001, Mann–Whitney test. **(C,D)** *myd88* or *rfp* KO larvae were injected with LPS. Quantification of NF-κB activity **(C)** and representative images **(D)** of the GFP intensity in the *Tg(NF*κ*B:GFP)* background in the indicated region (red box). Scale bar: 500 µm. Results are presented as mean ± SD (*n* = 3 independent experiments with over 20 larvae each/experiment). ****p < 0.0001, Kruskal–Wallis test. **(E)** Survival curve of *myd88* or *rpf* KO larvae injected with LPS. One representative experiment of three independent biological repeats (*n* = 20 each group) is shown. **p < 0.01, Gehan–Breslow–Wilcoxon test.

Myd88 is a critical adaptor protein mediating pro-inflammatory signaling downstream of Toll-like receptors *via* the activation of the NF-ĸB pathway (56). Although the LPS receptor in zebra fish is not clear (12), we next determined whether Myd88 is required for LPS-induced systemic inflammation. We utilized the CRISPR/Cas9 system to knock out *myd88* as previously described (25) and observed a decrease in baseline NF-κB activity in the uninjected larvae (Figures 5A,B). The disruption of *myd88* reduced NF-κB-induced GFP signal intensity in LPS-injected larvae at 8 and 24 hpi (Figures 5C,D), which coincides with significantly improved survival rates (Figure 5E), suggesting that MyD88 is a central mediator of inflammation downstream of the LPS receptor in fish, further confirming the conservation of zebra fish and humans (57).

### Global Proteomic Profiling in the Endotoxemia Model

**Figure 6:**
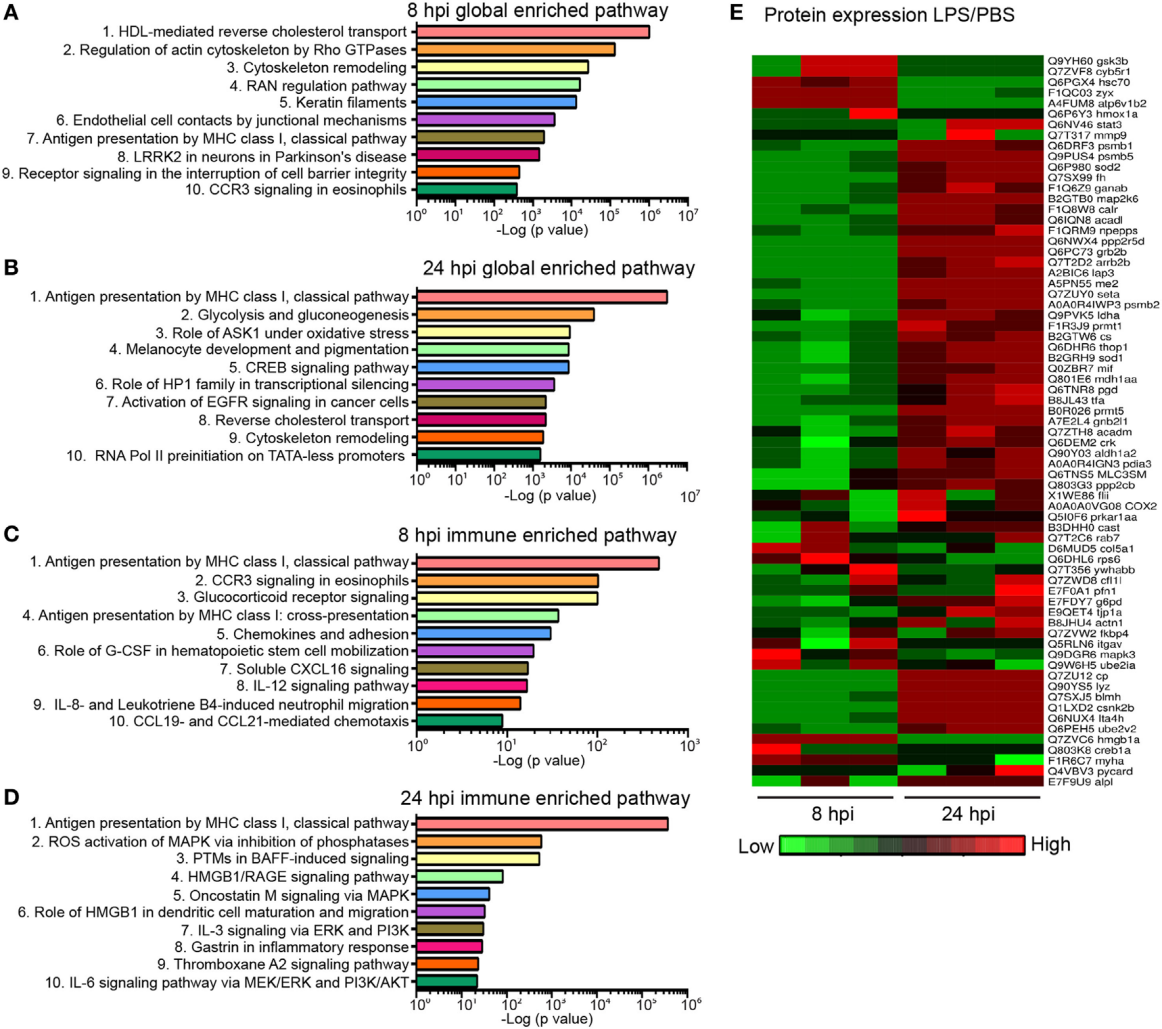
Global proteomics analysis in the zebra fish endotoxemia model. Three days post fertilization larvae were either injected with PBS or lipopolysaccharide (LPS). Samples were flash-frozen at 8 and 24 hours post injection (hpi) and subjected to proteomics analysis. Proteins with significant difference in expression levels were then subjected to pathway analysis. **(A,B)** Top 10 global-enriched pathways at 8 **(A)** and 24 hpi **(B)** in LPS-injected larvae compared to that of PBS-injected control. **(C,D)** Top 10 immune pathways enriched at 8 **(C)** and 24 hpi **(D)** LPS-injected larvae compared to that of PBS-injected control. The −log_10_ *p*-values below the graph were calculated by MetaCore software and indicate the magnitude of alteration of the whole network (*n* = 3 independent experiments with over 20 larvae each/experiment). **(E)** Fold changes of proteins involved in the top 10 enriched immune pathways at 8 and 24 hpi. The heat map presents the relative expression of proteins in LPS-injected larvae compared to that of PBS-injected control. Three independent experiments are shown.

Next, we sought to address whether the biological response to endotoxemia in zebra fish larvae would mimic that seen in humans. Larvae were injected with PBS, LPS, or left uninjected, followed by whole larvae proteomic analysis at 8 and 24 hpi. The proteomes of PBS-injected larvae and those of uninjected control were found to be similar (raw data uploaded, see section “Materials and Methods”). Proteins with significant (*p* < 0.05) and over 1.5-fold changes among the PBS- and the LPS-injected groups were converted to the human orthologs and subjected to MetaCore™ pathway enrichment analysis using the Uniprot database (Tables S2 and S3 in Supplementary Material). At 8 hpi, many of the enriched pathways were involved in cell mobilization and motility, cell junction alterations, as well as innate immune functions (Figure 6A), suggesting an acute activation of immune cells and inflammation in tissues during endotoxemia (18, 58). At 24 hpi, Creb-signaling pathways, reverse cholesterol transport, ROS, and Ask1 activation pathways were enriched, showing a more sustained biological response and pathway crosstalk (59–62) (Figure 6B). In immune/inflammation-related pathways (Figures 6C,D; full list in Table S4 in Supplementary Material), pathway enrichments of hematopoiesis and chemotaxis along with inflammation were seen at 8 hpi (Figure 6C). While at 24 hpi, Hmga/b, IL-3, and IL-6 response pathways were enriched, which are hallmarks of prolonged over-inflammation (Figure 6D) (63–65). To further investigate the dynamics of the individual proteins involved in the inflammation processes, a heat map was generated for both time points (Figure 6E). The upregulation of Gsk3b (66) and downregulation of Stat3 (67) during acute phases of endotoxemia were observed, consistent with their role in promoting inflammation. At 24 hpi, Stat3 and Sod1/2 (68) were upregulated possibly to facilitate resolution. Meanwhile, immune effector proteins such as Mif were upregulated, suggesting the persistence of systemic inflammation (69).

### Shp2 Inhibitor Suppresses Inflammation in the Endotoxemia Model

**Figure 7:**
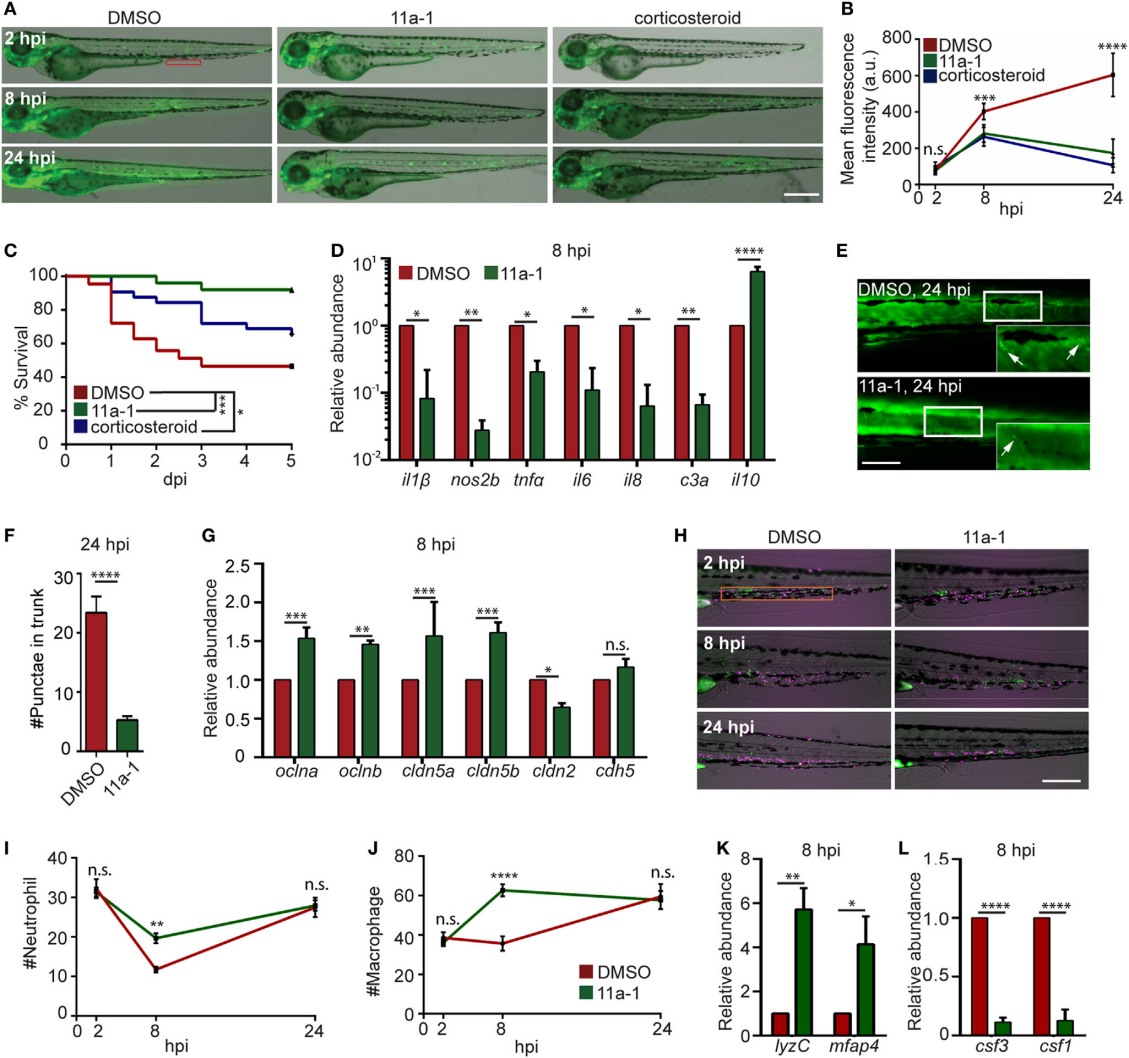
Shp2 inhibitor reduces lipopolysaccharide (LPS)-induced systemic inflammation and mortality. **(A,B)** Zebra fish embryos of *Tg(NF:*κ*B:GFP)* were treated with DMSO, Shp2 inhibitor (11a-1), or corticosteroids and injected with LPS. Representative images **(A)** and quantification **(B)** of the GFP intensity in the indicated region. Scale bar: 500 µm. Results are presented as mean ± SD **(D)** (*n* = 3 independent experiments with over 20 larvae each/experiment). ***p < 0.001, ****p < 0.0001, Kruskal–Wallis test. **(C)** Survival curve LPS-injected larvae treated with DMSO, 11a-1, or corticosteroid. One representative experiment of three independent biological repeats (*n* = 20 each group) is shown. *p < 0.05, ***p < 0.001, Gehan–Breslow–Wilcoxon test. **(D)** Transcript levels of genes encoding pro-inflammatory and anti-inflammatory cytokines in whole larvae treated with DMSO or 11a-1 at 8 hpi. Results were normalized with *ef1α* and are presented as means ± SD (*n* = 3 biological repeats with 20 larvae in each group). *p < 0.05, **p < 0.01, ****p < 0.0001, Sidak’s multiple comparisons test. **(E,F)** *Tg(*β*-actin:secANV-YFP)^pu17^* were treated with DMSO or 11a-1 and injected with LPS. Representative image **(E)** and quantification **(F)** of apoptotic cell puncta in the trunk. Scale bar: 200 µm. ****p < 0.0001, Mann–Whitney test. **(G)** Transcript levels of genes encoding vascular and cell junction proteins in whole larvae at 8 h post LPS injection treated with DMSO or 11a-1. Results were normalized with *ef1α* and are presented as means ± SD (*n* = 3 biological repeats with 20 larvae in each group). **p < 0.01, ***p < 0.001, Sidak’s multiple comparisons test. **(H–J)** *Tg(mpeg:H2B-GFP)* were crossed with *Tg(mpx:mCherry)*, treated with DMSO or 11a-1 and injected with LPS. Representative images **(H)** and quantification of neutrophil **(I)** and macrophage **(J)** numbers in the caudal hematopoietic tissue (orange box). One representative result of three independent experiments was shown (*n* = 20). Scale bar: 200 µm. Results are presented as mean ± SD (*n* = 3 independent experiments with over 20 larvae each/experiment). **p < 0.01, ****p < 0.0001, Mann–Whitney test. **(K,L)** Transcript levels of genes encoding *lyzC* and *mfap4* **(K)**, *csf3* and *csf1* **(L)** in whole larvae treated with DMSO or 11a-1 after LPS injection. Results were normalized with *ef1α* and are presented as means ± SD (*n* = 3 biological repeats with 20 larvae in each group). *p < 0.05, **p < 0.01, ****p < 0.0001, Mann–Whitney test.

To demonstrate the feasibility of using our model for drug discovery and mechanistic study, two different classes of pharmacological agents were evaluated. Corticosteroids have been extensively used for the suppression of inflammatory responses, including zebra fish studies (27, 70), where a mixture of hydrocortisone and dexamethasone was used (71). We also determined the effect of a previously published protein tyrosine phosphatase inhibitor, the Shp2 (Ptpn11a) inhibitor 11a-1 (28), in our endotoxemia model. Shp2 is required for pro-inflammatory cytokine production, ROS production, and macrophage M1 polarization (72, 73), but has not been tested as a drug target in endotoxemia. As expected, significant decreases in the NF-ĸB levels (Figures 7A,B) and mortality (Figure 7C) in LPS-injected larvae treated with either the Shp2 inhibitor or corticosteroid were noted. In 11a-1-treated larvae, inflammatory cytokines were decreased, whereas anti-inflammatory *il-10* was increased at 8 hpi (Figure 7D). Tissue damage was significantly reduced (Figures 7E,F), and the vascular junction genes were preserved at 8 hpi when treated with 11a-1 (Figure 7G). In addition, 11a-1 treatment also preserved the phagocyte numbers in the CHT (Figures 7H–J) as well as in the whole larvae (Figure 7K) and mitigated the increase of myelopoiesis-stimulating growth factors at 8 hpi (Figure 7L). Together, our finding that corticosteroids can suppress the LPS-induced inflammatory responses in the zebra fish endotoxemia model further validated its utility for the discovery of novel therapeutic agents to treat endotoxemia. The observed anti-inflammatory and protective effects by compound 11a-1 in our LPS-induced endotoxemia model suggest that Shp2 inhibition may provide a potential strategy for combating endotoxemia.

## Discussion

Here, we report a zebra fish endotoxemia model facilitated by IV injection of LPS into 3 dpf larvae. Hallmarks of acute endotoxemia including systemic inflammation, extensive tissue damage, loss of vascular junction integrity, immune cell mobilization, emergency hematopoiesis, and host mortality were observed herein. It is well known that during systemic inflammation, neutrophils and macrophages migrate out of the zebra fish CHT, enter the circulation and peripheral tissues, and perform immune functions to combat the immunostimulus (23, 74). With a single dose of LPS administration, emergency hematopoiesis happened quickly, and by 24 hpi, the immune cells in the CHT and the entire larvae were replenished. Therefore, our model provides a great tool in mechanistic studies of emergency hematopoiesis and resolution of the systemic inflammation. We have reiterated several characteristics of the resolution after systemic inflammation such as elevated *il-10* levels to shift the immune response to anti-inflammation (Figure 2D) (42), increased Occludin-5 which facilitate vascular tissue repair (Figure 3I) (49), upregulated hematopoiesis growth factors to replenish the immune reservoir (Figure 4H) (55), while also identifying many proteins associated with inflammation resolution with proteome analysis (Table S2 in Supplementary Material). With this model, we may be able to further our understanding on the resolution stages of acute endotoxemia.

We were able to recapitulate several previously reported changes in protein abundance associated with endotoxemia in our proteomic results (75, 76), including proteins and pathways involved in inflammation, vascular and organ damage, hematopoiesis, metabolism, inflammation resolution, and tissue repair (full list in Table S2 in Supplementary Material). For example, the aspartate aminotransferase Got2 was upregulated at advanced stages of endotoxemia which is closely correlated with liver damage (77, 78). Hmga2, which is often seen upregulated in human sepsis and which has been shown to be involved in immune activator functions and tissue damage, increased over time in our endotoxemia model (79, 80). Casein kinase II (CkII) has been reported to be an anti-inflammatory regulator required for anti-inflammatory cytokine production such as Tgf-β (81) and observed to be downregulated in multiple inflammatory diseases (82). Here, we observed, for the first time in animal models, an upregulation of CkII at the later stage of acute endotoxemia, providing supporting evidence that CkII possibly drives the initiation of the resolution response. Another family of proteins that we have linked to endotoxin immune response is the Karyopherin alpha family proteins which have been known to promote inflammation *via* p65 NF-κB activation (83). However, we were not able to detect differences in various immediate inflammatory proteins such as Nos2b and IL1b, but were detected to be upregulated at the mRNA level. This could be due to the stringent cutoff criteria of our proteomic studies (19).

While corticosteroids are often used in a broad spectrum of inflammatory conditions, they come with adverse side effects (84). As such, there is a constant search for specific drugs to treat specific conditions. Shp2 is the first reported oncogenic tyrosine phosphatase that regulates multiple cellular process, including signal transduction, downstream of growth factor receptor signaling, and Schwann cell development (85, 86). Multiple variants of Shp2 inhibitors are available for treating cancers (87), of which TNO155 synthesized by Novartis is currently in phase I clinical trial for lung and head and neck cancer (NCT03114319). In regard to innate immune-mediated systemic inflammation, Shp2 is an upstream activator of Akt and Ras pathways (88, 89) which promotes cardiac mitochondria dysfunction in sepsis mice models (90) and exacerbates systemic lupus erythematosus (91). Shp2 inhibition promotes innate immune cells to shift toward an anti-inflammatory phenotype with more IL-10, Stat3 production, and an M2-dominant macrophage phenotype (73, 92). Here, we utilized a recently developed Shp2 inhibitor 11a-1 (28) and found drastically reduced systemic NF-ĸB and immune activation with decreased lethality in treated larvae, suggesting a potential role of the Shp2 inhibitor as an anti-inflammatory drug. Although the mechanisms of how Shp2 promotes systemic inflammation in the zebra fish model requires further investigation; this proof of concept demonstrates that our endotoxemia model can be used to test candidate drugs at a whole organism level.

Though LPS has been widely used as an immunostimulant, in many fields of studies, the response and tolerance toward LPS vary greatly between species and is also dependent on the routes of administration. Humans have a much lower tolerance than other commonly used model animals (93, 94). It is speculated that mice have a much higher tolerance to LPS due to their innate immunoglobulins against LPS. LPS-antibody complexes signal through Fc receptors, enhancing the clearance of LPS by recruited phagocytes (95) or suppressing the signals from TLRs (96) which results in divergence from the response in humans (6). Zebra fish larvae are also more tolerant to LPS compared to humans (17), possibly due to their aquatic habitats (8) or the identified TLR4 delivering an inhibitory signaling (97). In contrast with the murine models, zebra fish larvae younger than 2 weeks do not have a functional adaptive immune system. These differences may be the reason why LPS-induced signaling pathway changes are not entirely conserved among the three species, highlighting the necessity of including multiple animal models. Another very interesting observation is that immunological priming by commensal at early developmental stage shapes inflammatory reactions later on (98). Future experiments using germ-free technique can be used to determine the effect of the immune priming in our endotoxemia model.

In summary, we provide here an endotoxemic model utilizing the strength of zebra fish larvae which faithfully represents the hallmarks and signatures of acute endotoxemia. We further show that our model can be genetically manipulated to study inflammation and treated with drugs to assess the effect of potential compounds in an endotoxemic scenario. It is our expectation that our newly developed zebra fish endotoxemia model will provide a complementary tool for a full understanding of acute inflammation in humans.

## Ethics Statement

This study was carried out in accordance with the recommendations of “Use of Zebrafish in the NIH Intramural Research Program.” The Animal Care and Use Protocol was approved by The Purdue Animal Care and Use Committee (PACUC) (Protocol number: 1401001018).

## Author Contributions

AH and QD conceived the project. AH, TG, SZ, NM, and CC performed experiments and analyzed data. TS performed and analyzed the proteomics data. SZ and Z-YZ provided essential reagents. AH and QD wrote the manuscript. All authors read and approved the final manuscript.

## Conflict of Interest Statement

The authors declare no competing commercial or financial interests.

## Supplementary Material

Movie S1 Circulation defects of LPS injected larvae at 8 hpi. Representative movie of the circulation in the caudal artery and vein of PBS injected (top) or LPS injected (bottom) larvae at 8 hpi. Red arrows: obstructed circulation. Scale bar: 100 µm.

Movie S2 Circulation defects of LPS injected larvae at 24 hpi. Representative movie of the circulation in the caudal artery and vein of PBS (top) or LPS injected obstructed (middle) and recovered (bottom) larvae at 24 hpi. Red arrows: obstructed circulation. Scale bar: 100 µm.
