# Supplementary material for: Development and Characterization of an Endotoxemia Model in Zebra Fish
Source: Front Immunol. 2018 Mar 29;9:607. doi: 10.3389/fimmu.2018.00607 (PMC5884884; doi:10.3389/fimmu.2018.00607)
Supplement: Supplementary file 3 [file Data_Sheet_1.docx]

**Supplementary information**

**
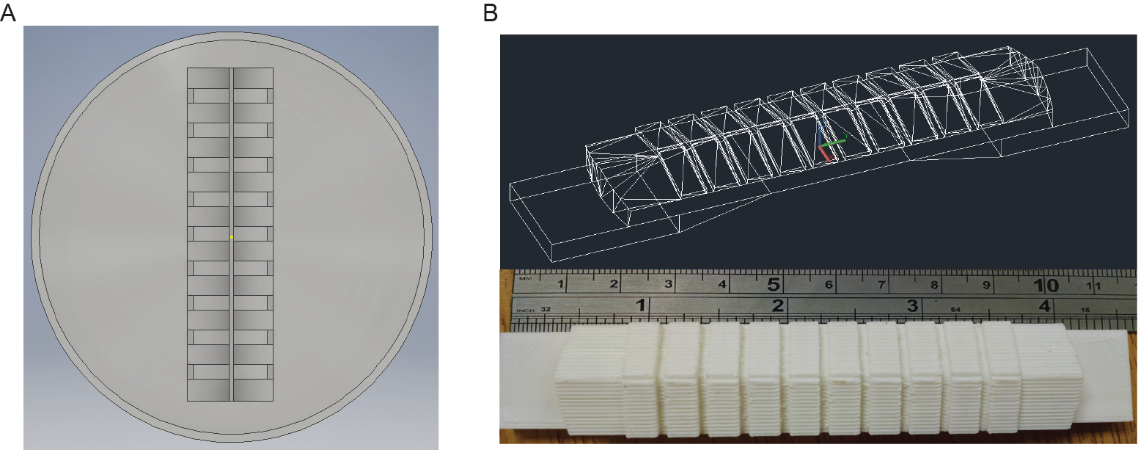
**

**Supplementary Figure 1. Schematic of Zebrafish intravenous injection plate.**

(A) Overlooking view of zebrafish intravenous injection plate. (B) AutoCAD and a representative image of the mold used to make the injection plate from a 10 cm dish.

**
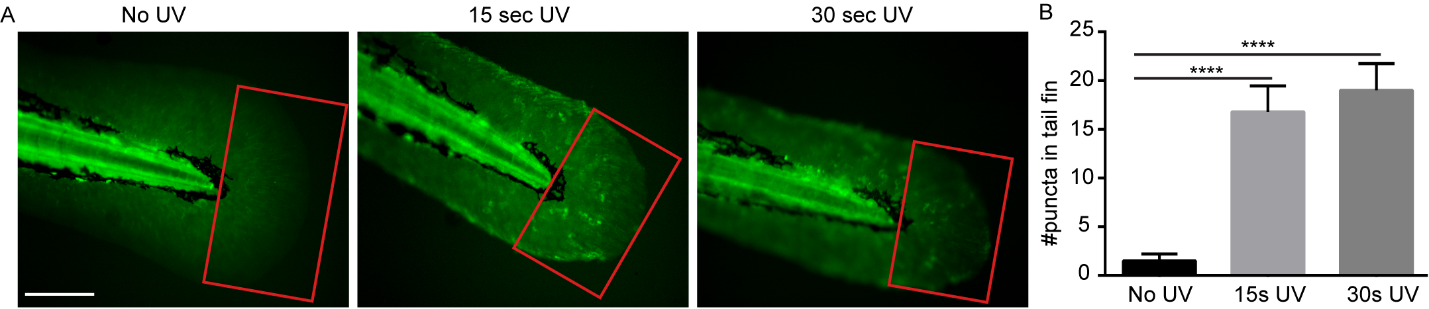
**

**Supplementary Figure 2. Characterization of *Tg(βactin:secA5-YFP)* line.**

(A) *Tg(βactin:secA5-YFP)* larvae at 3 dpf were subjected to either no UV, 15 sec, or 30 sec irradiation. (B) Quantification of puncta in the boxed region in tail fin. Scale bar: 100 µm. Results are presented as mean ± s.d. (N = 3 independent experiments with over 20 larvae each/experiment). ****, p<0.0001, Kruskal-Wallis test.

**Table S1. List of primers used for RT-qPCR**

**Table S2. List of proteins with significant changes in expression levels upon LPS injection.**

Full list of proteins with significant (p<0.05) and over 1.5 fold change when comparing LPS injected to PBS injected groups. Tabs are organized for 8 hpi and 24 hpi respectively. Full dataset is uploaded to MassIVE database.

**Table S3. List of significantly altered pathways upon LPS injection.**

Proteins listed in Table S2 were subjected to MetaCore pathway analysis for total pathway enrichment analysis. A list of total enriched pathways at 8 hpi and 24 hpi are shown in individual tabs.

**Table S4. List of significantly altered immune and inflammation pathways upon LPS injection.**

Proteins listed in Table S2 were subjected to MetaCore pathway analysis for immune and inflammation related pathway enrichment analysis. A list of total enriched pathways at 8 hpi and 24 hpi are shown in individual tabs.

**Movie S1. Circulation defects of LPS injected larvae at 8 hpi.**

Representative movie of the circulation in the caudal artery and vein of PBS injected (top) or LPS injected (bottom) larvae at 8 hpi. Red arrows: obstructed circulation. Scale bar: 100 µm.

**Movie S2. Circulation defects of LPS injected larvae at 24 hpi.**

Representative movie of the circulation in the caudal artery and vein of PBS (top) or LPS injected obstructed (middle) and recovered (bottom) larvae at 24 hpi. Red arrows: obstructed circulation. Scale bar: 100 µm.
